# Examining the Role of Attention and Sensory Stimulation in the Attentional Repulsion Effect

**DOI:** 10.3389/fpsyg.2019.00238

**Published:** 2019-02-12

**Authors:** Anna M. Petersson, Matthew D. Hilchey, Jay Pratt

**Affiliations:** ^1^Department of Psychology, Simon Fraser University, Burnaby, BC, Canada; ^2^Department of Psychology, University of Toronto, Toronto, ON, Canada

**Keywords:** space-based attention, spatial vision, visual perception, attentional repulsion effect, visual attention

## Abstract

It has been suggested that visual attention warps space, such that stimuli appearing near its locus are perceived as farther away than they actually are. This is known as the attentional repulsion effect (ARE). Recent data challenge the role of attention as the sole factor responsible for the ARE, suggesting instead that the ARE is, at least in part, a product of low level sensory interactions between a peripheral orienting cue and the Vernier target stimulus used to measure the effect. Here, we directly test whether attentional orienting, without a cue in peripheral vision to guide attention, is sufficient for generating an ARE. In Experiment 1, attention was guided to the visual periphery by a central symbolic cue that reliably indicated the locations of to-be-identified targets in peripheral vision. On a subset of trials, we probed for an ARE with Vernier targets. Reaction time (RT) data revealed that the cue guided attention but there was no trace of an ARE. In Experiment 2, we ensured that the Vernier targets were sensitive to the ARE by using the standard spatially uninformative peripheral cue to guide attention instead of the central symbolic cue. RT data again revealed that the cue guided attention, while the Vernier targets revealed an ARE. Collectively, these data suggest that attentional orienting without peripheral sensory stimulation is not sufficient for generating an ARE.

## Introduction

Traditionally, attentional orienting is inferred from chronometric measurements in basic cueing studies (e.g., Posner et al., 1980; Posner and Cohen, 1984). Over the years, these methods have been refined to show that relatively salient properties of visual stimuli (e.g., motion onset, color singletons, looming, abrupt visual onsets and offsets, and relatively high or low contrast differences) generally improve the efficiency of signal detection in their vicinity (e.g., Takeuchi, 1997; Reynolds et al., 2000; Pratt and McAuliffe, 2001; Abrams and Christ, 2003; White et al., 2014). This stimulus-driven, or involuntary, form of attentional orienting is usually inferred when reaction times (RTs) are fast to targets appearing at the location of a prior salient stimulus relative to elsewhere. In the absence of relative physical salience to attract attention, attention can be guided by symbolic stimuli, appearing in central vision, that reliably predict the locations of upcoming target stimuli in peripheral vision (e.g., Jonides, 1981; Ristic and Kingstone, 2006). This voluntary, or associative, form of attention also results in RTs that are fast to targets appearing at predicted relative to unpredicted regions (see Klein, 2009; Awh et al., 2012, for reviews). Simply put, the major difference between these forms is that involuntary orienting is pulled directly to the location of a task-irrelevant but salient sensory event whereas voluntary orienting is pushed to the location of a prospectively meaningful event.

Interestingly, departures from traditional chronometric methods have revealed that attentional orienting may also fundamentally alter how stimuli are perceived in space. One example of this is the Attentional Repulsion Effect (ARE). This refers to the finding that stimuli are judged as appearing further away from an attended location than they actually are (Suzuki and Cavanagh, 1997). In a typical ARE paradigm, attention is presumed to be oriented to quadrants in the visual field by abrupt visual onset stimuli, or cues, that suddenly appear and disappear (also referred to as “onset-offset” cues; Pratt and Arnott, 2008). Shortly thereafter, a Vernier stimulus appears, which consists of two parallel, or nearly parallel, line segments – one above and the other below a central fixation stimulus. The top line of the Vernier stimulus is judged as either offset to the left or right of the bottom line with a forced left/right key-press response. The ARE is inferred from the observation that there is a response bias in the direction opposite the cue, indicating that participants perceive the top Vernier as further away from the cued location than it actually is.

The ARE peaks at cue-target onset asynchronies (CTOAs) between 100 and 200 ms. Suzuki and Cavanagh (1997) interpreted this temporal profile as evidence that the effect was attentional in nature, as they noted that a chronometrically-measured involuntary attentional cuing effect would be expected to fall within this time frame. This is due to the fact that a relatively short CTOA would not provide sufficient time for attention to disengage the cued location prior to target appearance, whereas a relatively long CTOA would allow attention to disengage the cued location prior to target appearance. In light of this, Suzuki and Cavanagh reasonably presumed that their peripheral cues were capturing attention. Further experimental results obtained by Suzuki and Cavanagh that bolstered the conclusion that the ARE is attentional include the findings that the ARE neither appeared to be a result of figural aftereffects nor apparent motion.

To examine whether the ARE could also be found with voluntary shifts of attention, Suzuki and Cavanagh (1997) performed an additional experiment that featured two diagonal pairs of cues differing in shape. Participants were instructed to attend to one of the shapes while ignoring the other. The results showed that, at CTOAs above 120 ms, the attended cues, as compared to the unattended cues, generated an ARE. Furthermore, in contrast to the involuntary-attention-induced effect, Suzuki and Cavanagh observed that this voluntary-attention-induced ARE occurred fairly independently of cue-Vernier CTOAs beyond 120 ms and could, in fact, be observed at CTOAs as long as 1500 ms. Nevertheless, both the voluntarily and involuntarily induced ARE decreased with increased exposure duration of the Vernier stimulus itself, with the effect dropping by more than half with 100–200 ms exposure durations. Suzuki and Cavanagh thus concluded that attention is germane to the ARE.

Further support for the ARE as an attentional phenomenon comes from Pratt and Arnott (2008), who examined whether AREs are expressed in various conditions that are known to induce attentional orienting. In short, the authors reasoned that if AREs ebb and flow with manipulations known to increase and decrease traditional chronometrically-measured attentional orienting effects, then it would be reasonable to infer that the ARE is dependent, at least in part, on attentional processes. As such, Pratt and Arnott used multiple different cue types to elicit AREs: abrupt onset cues (i.e., cues that appeared onscreen and remained on screen), abrupt offset cues (i.e., cues that initially were present onscreen and then disappeared), abrupt onset-offset cues (i.e., cues that appeared and then disappeared), and pop-out cues (i.e., where four cues appeared, one in each quadrant, but one cue was in a distinct color). Assessing the magnitude of the ARE in response to these various cues revealed a pattern comparable to that expected from RTs in prior cueing studies. That is, in conditions in which RT typically does not differ across cue types, the magnitudes of the AREs were identical. In addition, when onset cues were pitted against offset cues, onset cues produced a larger ARE than did offset cues, again mimicking the pattern seen for RT advantages during such conditions. Finally, a comparison between onset-offset cues and pop-out cues revealed that the former produced a larger ARE than the latter, just as the former types of cues typically produce a larger RT advantage than the latter.

Yet, other studies involving the ARE have generated results that appear to be inconsistent with these attentional attributions. For example, DiGiacomo and Pratt (2012) examined the ARE under monocular cueing conditions (cues and targets presented to a single eye) and whether it survives interocular transfer (i.e., cues presented to one eye and targets presented to the other). As binocular vision (i.e., cues and targets presented to both eyes) is processed in higher-order areas of the extrastriate cortex (visual areas V2–V5), a failure to find an ARE during a monocular experimental condition would suggest that the ARE is generated higher up in the visual processing pathway (i.e., within visual processing areas beyond V1). However, the authors found that their monocular condition generated an ARE similar to the binocular condition, which signifies lower level visual processing. Furthermore, in another experiment, DiGiacomo and Pratt made use of visual occlusion goggles, and were thus able to present the cue to one eye of their participants while presenting the subsequent Vernier-target to the other eye. If the ARE arises due to mechanisms of attentional orienting, as suggested by Suzuki and Cavanagh (1997), one would predict interocular transfer would occur and that the ARE would persist under such conditions. Yet, the ARE was lost during this experiment, indicating that it occurs at a stage during visual processing prior to interocular information transfer.

Findings from Gozli and Pratt (2012) also cast doubt on the attentional nature of the ARE. In one experiment, the Vernier stimulus comprised ten lines, five above and five below fixation. One target line above and below fixation was red, whereas the remaining lines were green. People judged whether the top red line was to the left or right of the bottom red line. Importantly, two onset-offset cues, either matching (e.g., red) or mismatching (e.g., green) the target Vernier color, preceded the Vernier stimulus on the diagonals of the display. In principle, this design leads to a color-based attentional control setting for ‘red’, meaning that only cues that match the target ought to capture attention. As an ARE was obtained regardless of whether the cue color matched the target color, the authors concluded that the ARE is resistant to top-down attentional control. A second experiment, in which the Vernier stimulus comprised heterogeneous colors and in which the color cues were accompanied by dimmer gray cues on the opposite diagonal, also demonstrated that matching and mismatching cues generated AREs. These mismatching cues would simply not be expected to capture attention, at least not according to standard chronometric indices (e.g., Folk et al., 1992; Bacon and Egeth, 1994). Taken together, these results demonstrate that the ARE may not be as exclusively dependent on attention as previously thought.

At this point, all prior studies on the ARE have generated the effect with peripheral cues, whether auditory (e.g., Arnott and Goodale, 2006) or visual (e.g., Suzuki and Cavanagh, 1997; Pratt and Turk-Browne, 2003; Pratt and Arnott, 2008; Kosovicheva et al., 2010; Fortenbaugh et al., 2011). Since the available evidence points toward the ARE as being, at least in part, a product of very low-level sensory interactions (DiGiacomo and Pratt, 2012), it now seems particularly important to determine whether the ARE can be observed when orienting is elicited without any peripheral cues. It is also interesting to keep in mind that no prior research on the ARE, with the exception of one experiment by Suzuki and Cavanagh (1997), has corroborated whether the cues used to induce the ARE were capturing attention, which is traditionally inferred from chronometric measurements. Although the peripheral cues were presumed to orient attention, there is no guarantee that this occurred. Simply, peripheral cues typically generate much weaker temporal effects when they occur in spaces that never contain target stimuli (e.g., Hilchey et al., 2012), which is typical of ARE experiments. In principle, the reason for this is that people pay less attention to information that occurs suddenly in places that are irrelevant. Our primary objective here was to thus determine whether the ARE can be induced with voluntary orienting in the absence of peripheral cues, while also corroborating the presence of orienting with standard chronometric measurements.

To achieve our objectives, we modeled our experimental design off of a task used by Van der Stigchel and Theeuwes (2007) which examined the relationship between voluntary orienting and saccadic curvature. In this study, the location of an eventual to-be-discriminated peripheral target was reliably cued (2/3 valid) with a line segment in central vision, which pointed to the upper left or right visual field. As expected, manual RTs to the target were faster for validly as compared to invalidly cued trials. On a smaller subset of trials (1/6), a beep occurred instead of a target, which signaled that an eye movement had to be made to a location in-between the two possible target locations. The trajectory of the eye movement deviated away from the cued location. In this manner, the authors were able to verify the presence of attentional orienting in the context of saccade curvature, similar to how we were trying to verify the presence of attentional orienting in the context of the ARE.

In our first task, prior to each experimental block, participants were instructed to orient attention, without making an eye movement, to the location arbitrarily indicated by a number cue (e.g., Ristic and Kingstone, 2006). The number cues were presented at fixation and reliably (55% valid across all trials; with chance being 33%) predicted the location of a to-be-discriminated target stimulus. The numbers selected for the cueing tasks were a “2” and a “5”, made from identical line segments, in order to ensure that the near left and right visual fields contained equal amounts of sensory stimulation. Keeping the distance on the number line between the two number cues small also minimized the risk of any possible shifts of attention due to processes influenced by the magnitude of the number (i.e., a SNARC effect; Dehaene et al., 1993). The CTOA was set to 500 ms in order to allow for the slower buildup of a voluntarily-attention-induced repulsion effect reported by Suzuki and Cavanagh (1997), as well as in order to maximize the attentional orienting effect observed by Ristic and Kingstone (2006). On a subset of trials, a Vernier stimulus appeared instead of the to-be-discriminated target stimulus, and the location of the top Vernier line had to be judged relative to the bottom line.

If attentional orienting is sufficient for the ARE to occur, then it should be possible to generate an ARE with these number cues, even though they do not directly stimulate the periphery. In contrast, if the ARE is more closely related to low-level sensory interactions, or peripheral stimulation, then the number cues should fail to generate an ARE. In any event, the number cues should show the standard chronometric index of an orienting effect, with faster responses to targets at validly as compared to invalidly cued locations.

## Experiment 1

To test whether attentional orienting is sufficient for the ARE in the absence of peripheral cueing, we used spatially informative numbers at fixation to cue attention to the visual periphery. We simply instructed our participants to allocate attention covertly to the locations indicated by the cues and then tested for the ARE, whilst verifying the presence of attentional orienting chronometrically.

### Materials and Methods

#### Participants

Twenty-six undergraduate students from the University of Toronto enrolled in first or second-year psychology courses successfully participated in the experiment in exchange for course credit. All participants had normal, or corrected-to-normal, vision and were naïve to the purpose of the experiment. Each participant provided informed consent prior to participating.

#### Apparatus and Stimuli

All experimental procedures were carried out on Dell computers projecting to 17” cathode ray tube (CRT) monitors, with 60 Hz refresh rates and screen resolutions of 1024 × 768, running inside a dimly lit, sound-attenuated room. Viewing distance was held constant at 50 cm with a chin-rest.

All stimuli were presented in white on a black background. The initial display consisted of a white fixation cross (+; 0.30° × 0.30° of visual angle) in the center of the display. The trial was initialized with a spacebar response. Seven hundred and fifty milliseconds later, the fixation cross was replaced by a number cue (2 or 5; subtending 1.00° × 1.00°) formed from straight-lined segments, that was centered in the display and that remained onscreen for 500 ms. The meaning of the cue was counterbalanced. For half of the participants, “2” and “5” predicted targets on the left and right of fixation, respectively, whereas for the other half the cues had the reverse meaning. Participants were told to covertly attend to the location indicated by the number cue because it reliably predicted the target location. Immediately after the cue vanished, the target appeared.

**FIGURE 1:**
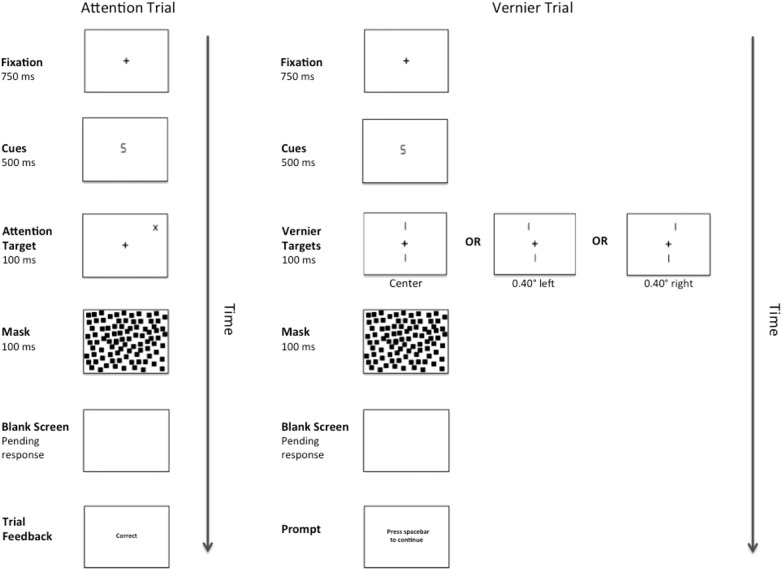
Example of a complete trial sequence for Experiment 1. The image is presented in reverse contrast and is not drawn to scale. The cues consisted of either a number “2” or a number “5”, and the Attention Targets (a “+”or an “x”) would appear in either the validly cued location or the invalidly cued location. Alternatively, after cue presentation, one of three versions of a Vernier Target (left, center, right) would appear in the center of the display. Following a pattern mask and pending the participant’s response, feedback in the form of the messages “correct” or “error” would appear during the Attention Trials and a message prompting the participant to press the spacebar to continue would appear during the Vernier Trials.

There were two types of target stimuli: Vernier Targets and Attention Targets. Attention Targets were “+” or “x” symbols (1.00° × 1.00°). These targets appeared diagonally 7.07° from fixation in either the top left or top right quadrant of the visual field and their locations were predicted by the number cues. The Vernier Targets consisted of two line segments, each subtending 1.40°. The bottom line was always centered 3.4° below fixation, whereas the top line was randomly either 3.4° above fixation, or above fixation and shifted either 0.40° to the right or left. Each target appeared onscreen for 100 ms. The target was followed by a pattern mask consisting of 1000 small squares (each measuring 0.25° × 0.25°) that formed an imaginary box (7.00° × 7.00°) centered on fixation. The pattern mask remained onscreen for 100 ms and was followed by a blank screen until a response was made or 2300 ms elapsed. A complete trial sequence for Experiment 1 appears in Figure 1.

#### Procedure

Participants were provided with oral instructions prior to the experiment. These instructions emphasized the importance of maintaining gaze on fixation whilst covertly orienting attention to the location indicated by the number cue. For attention targets, participants were instructed to respond to the targets as quickly and accurately as possible by pressing one of two pre-specified keys on the keyboard: “z” and “/” keys for “x” and “+” targets, respectively. The cue predicted the target location 80% of the time on trials containing attention targets. For Vernier targets, the participants were instructed to indicate whether the top Vernier line was displaced to the left or right relative to the bottom Vernier line by pressing the “z” or “/” key, respectively. The cue predicted the target location 0% of the time on trials containing Vernier targets. Each experimental session consisted of 32 practice trials immediately followed by 516 experimental trials, of which 156 and 360, respectively, were Vernier and attention target trials. Thus, for the experimental trials, the overall probability that a target was validly cued was 55.81%.

During the experiment, the participants were given performance feedback for the Attentional trials in the form of one-word messages displayed at fixation (“correct” or “error”). After the response in a Vernier trial, the message “press spacebar to continue” appeared. If a trial timed-out, the message “too slow” appeared. In addition, if a response was provided prior to the target presentation the message “too soon” appeared. All feedback was acknowledged by pressing the spacebar. The study was approved by the research ethics board at the University of Toronto (#32884; Binding across perception and action).

#### Data Analysis

Data from seven participants were excluded and replaced because performance was at chance levels or worse on Vernier trials in which the top line was left or right of center, collapsing across levels of cueing. These exclusions were not the result of inordinately large AREs (i.e., left cue leads to right response and right cue leads to left response, regardless of Vernier Type), which would also yield chance performance; these participants were instead at, or below, chance levels even when the ARE would have improved performance. These participants either guessed (*n* = 3) or were strongly biased toward making the same response to every Vernier type (*n* = 4), leading to generally flat psychometric functions. An additional participant was excluded and replaced due to an extraordinarily large RT advantage for Attention Targets at the cued location^1^. A final participant was excluded and replaced because they failed to respond within the response window on 48% of the trials. An additional analysis that included 8 of the 9 dropped participants (the participant who often failed to respond within the allotted time was not included due to the low amount of available data) revealed the same pattern of results, albeit unsurprisingly the effect of the Vernier Target became weaker (as these participants were either unable or unwilling to discriminate the Vernier Target as note above).

Only experimental trials were included in the analysis. Trials on which a response was made before the target (0.04%) or on which a response was not made within the 2.5 s target response window (0.6%) were excluded from analysis. For the RT analysis below, all Attentional trials that contained errors (6.5%) were removed. Next, z-scores were computed from the RTs for each participant at each level of cueing. Z-scores greater than 3 (1.67% of the correct RT trials) were considered outliers and excluded from analysis.

### Results and Discussion

#### Attention Targets

**FIGURE 2:**
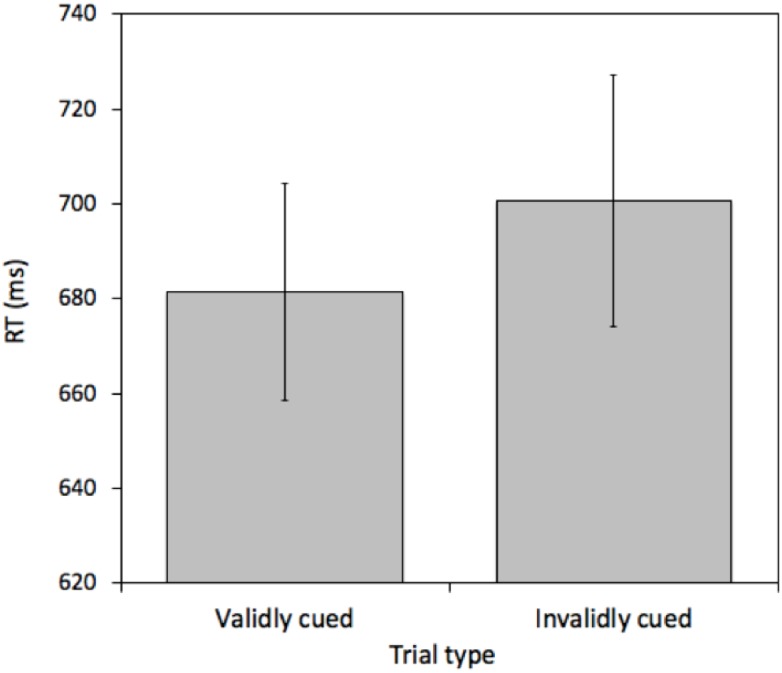
Mean reaction times (RTs) measured in milliseconds (ms) for validly and invalidly cued Attention Trials for Experiment 1. Error bars represent the standard error of the mean.

A pairwise *t*-test comparing mean RTs between validly cued and invalidly cued targets revealed a reliable effect [*t*(25) = 2.575, *p* = 0.016, *M* = 19.26 ms, 95%CI = 3.86 – 34.66 ms]. Mean RTs were faster on validly (681.43 ms) relative to invalidly (700.69 ms) cued trials (see Figure 2). This effect was not due to a speed-accuracy tradeoff (valid cue error rate = 6.45% and invalid cue error rate = 6.69%).

#### Vernier Targets

**FIGURE 3:**
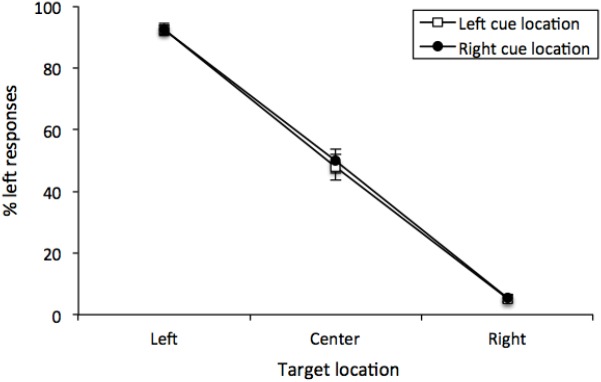
Mean percentage of “left” responses for Vernier Trials during Experiment 1. This figure illustrates the mean percentage of “left” responses given by the participants at each of the possible three Vernier Target display locations (left, center, and right). Error bars represent the standard error of the mean.

The percentage of “left” responses was computed for each participant for each combination of Cue Location (left or right) and Vernier Target (top line left, center or right of bottom line). These data were analyzed with a 2 (Cue Location) × 3 (Vernier Target) repeated-measures analysis of variance (ANOVA). There was a main effect of Vernier Target [*F* (2, 50) = 370.95, *MSE* = 266, *p* < 0.001, ${\text{η}}_{\text{G}}^{2}$ = 0.8771], but neither a main effect of Cue Location [*F*(1, 25) < 1] nor any interaction [*F*(2, 50) < 1]. Thus, participants were able to distinguish between the different Vernier Targets, and Cue Location did not have any effect on performance (see Figure 3). This was confirmed by examining only center Vernier targets, which did not show an effect of Cue Location [*t*(25) = 0.733, *p* = 0.470, *M* = 1.96%, 95%CI = -7.49 – 3.56%].

Despite evidence of voluntary orienting, no ARE emerged in Experiment 1. These data simply suggest that AREs depend critically on the appearance of peripheral cues.

## Experiment 2

There are certain aspects of our first experiment that may cast some doubt on whether attention is insufficient for generating an ARE. In principle, Experiment 1 presented a dual-task scenario by unconventionally intermixing attention targets with Vernier targets in order to verify the presence of orienting chronometrically. Conceivably, however, participants may have prioritized one task over the other, artificially leading to a divergence between the temporal measurement of orienting and the spatial ARE. To help ensure that it is possible to obtain an ARE in this dual-task scenario, we considered it prudent to conceptually replicate the ARE results obtained by Suzuki and Cavanagh (1997) in the dual-task of Experiment 1, except here we reverted to the standard approach for generating an ARE by (1) using a typical, spatially uninformative abrupt onset-offset cue to induce it, and (2) reducing the temporal interval between the cue and target in order to accommodate the faster buildup of an exogenously-generated ARE (Suzuki and Cavanagh, 1997).

### Materials and Methods

#### Participants

In order to match the original number of participants from the first experiment, twenty-six undergraduate students from the University of Toronto enrolled in first or second-year psychology courses successfully participated in the experiment in exchange for course credit. All participants had normal, or corrected-to-normal, vision and were naïve as to the purpose of the experiment. None of the participants had previously participated in Experiment 1 and each participant provided informed consent prior to participating.

#### Apparatus and Stimuli

Identical to Experiment 1, except the fixation cross did not transform into a number cue and was instead replaced by an abrupt onset-offset cue (a filled white circle with a radius of 0.25°) at one of the two possible Attention Target locations.

#### Procedure

**FIGURE 4:**
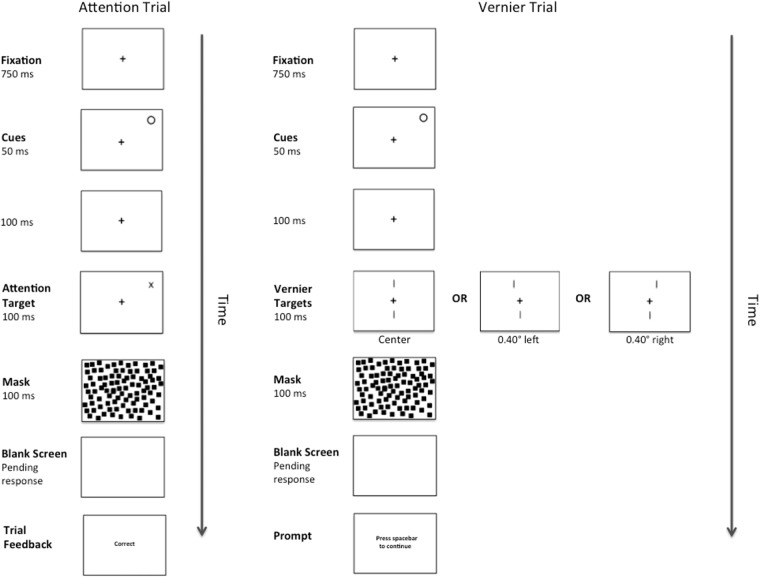
Example of a complete trial sequence for Experiment 2. Timing is displayed in milliseconds (ms) and image is presented in reverse contrast. The trial sequences in Experiment 2 were identical to those presented in Experiment 1, with the exception of an abrupt onset-offset cue appearing randomly at one of the two possible Attention Target locations for 50 ms followed by a temporal interval of 100 ms (total CTOA 150 ms) prior to the presentation of an Attention or Vernier Target. Following a pattern mask and pending the participant’s response, feedback in the form of the messages “correct” or “error” would appear during the Attention Trials and a message prompting the participant to press the spacebar to continue would appear during the Vernier Trials.

A complete trial sequence for Experiment 2 appears in Figure 4. This was identical to Experiment 1, except for that the abrupt onset-offset cue appeared randomly at one of the two possible Attention Target locations for 50 ms. One hundred ms after the offset of the cue (total CTOA = 150 ms), either an Attention or Vernier Target appeared. As is typical with such peripheral cues, participants were instructed to ignore the cue and were correctly informed that it did not predict the eventual target location. As before, there were 156 Vernier Target trials. The number of Attention Target trials was reduced to 312 to make the cue unpredictive of target location.

During pilot testing, we realized that participants found Vernier Target trials more difficult in Experiment 2 than in the prior Experiment 1. To help participants along, they first practiced Attention Target and then Vernier Target trials in different blocks. During these practice blocks, the two lines of the Vernier stimulus were always offset and error feedback was always provided. Each practice block consisted of 16 practice trials. Upon completion of the practice blocks, the experiment began. As before, the experiment was preceded by 32 general practice trials and Attention and Vernier Target trials were randomly intermixed. The study was approved by the research ethics board at the University of Toronto (#32884; Binding across perception and action).

#### Data Analysis

Data from ten participants were excluded and replaced because performance was at chance levels or worse on Vernier trials in which the top line was left or right of center, collapsing across levels of cue location. As before, these participants were either unable or unwilling to judge the relative placement of the top line. Again, this was not the result of an inordinately large ARE, and their psychometric functions were generally flat, with two exceptions: One participant was near perfect when the top line of the Vernier stimulus was shifted to the right, but at chance when it was shifted to the left; the other participant showed the opposite pattern. Regardless of whether the participants were excluded or not, the results remained the same.

Only experimental trials were included in the analysis. Trials on which a response was made before the target (0.07%) or on which a response was not made within the 2.5 s target response window (0.33%) were excluded from analysis. For the RT analysis below, all Attentional trials that contained errors (6.67%) were removed. Next, z-scores were computed from the RTs for each participant at each level of Cueing. Z-scores greater than 3 (1.88% of correct RT trials) were considered outliers and excluded from analysis.

### Results and Discussion

#### Attention Targets

**FIGURE 5:**
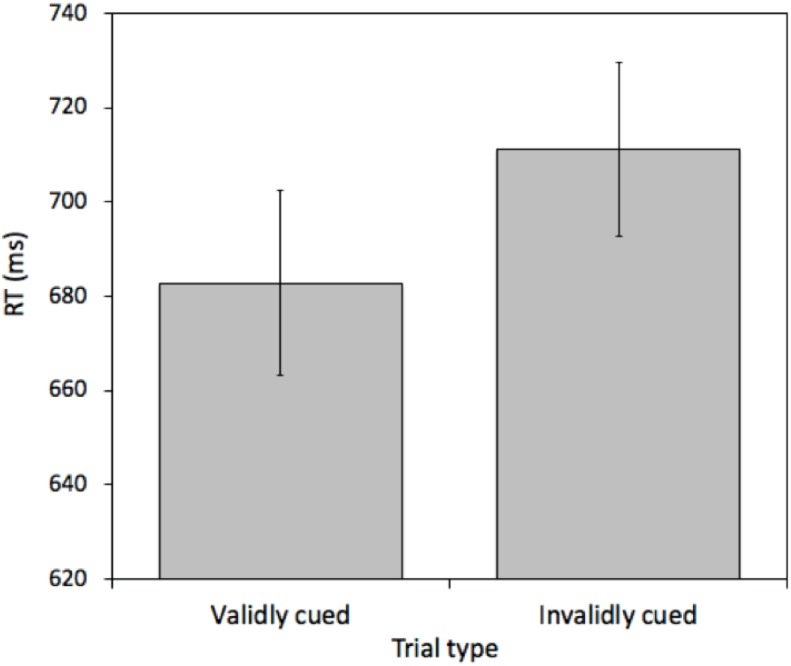
Mean reaction times (RTs) measured in milliseconds (ms) for validly and invalidly cued Attention Trials for Experiment 2. Error bars represent the standard error of the mean.

A pairwise *t*-test comparing mean RTs between validly cued and invalidly cued targets revealed an effect [*t*(25) = 4.968, *p* < 0.001, *M* = 28.31 ms, 95%CI = 16.57 – 40.03 ms]. Mean RTs were faster on validly (682.77 ms) relative to invalidly (711.07 ms) cued trials (see Figure 5). This effect was not due to a speed-accuracy tradeoff (valid cue error rate = 6.43% and invalid cue error rate = 6.93%). Furthermore, importantly, the magnitude of this cueing effect was statistically indistinguishable from the magnitude of the cueing effect in Experiment 1 [*t*(50) = 0.9618, *p* = 0.34, *M* difference = 9.04 ms, 95%CI of *M* difference = -9.84 – 28.30 ms].

#### Vernier Targets

**FIGURE 6:**
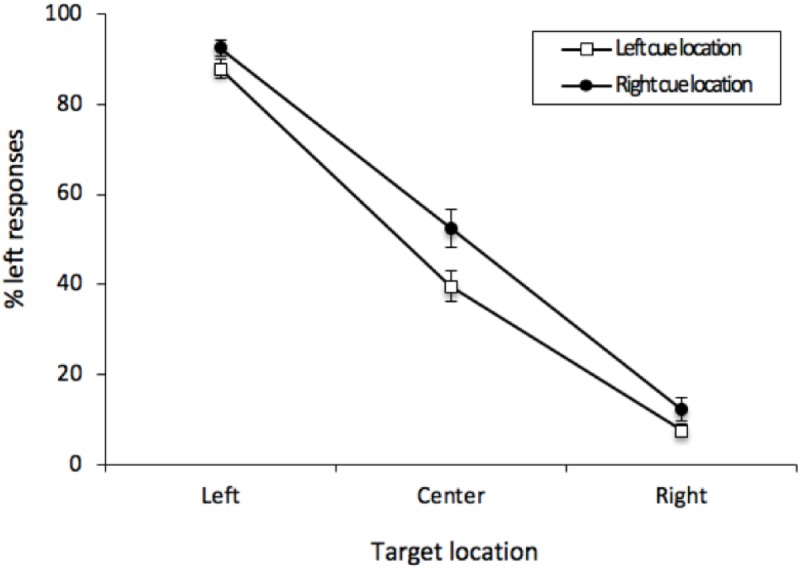
Mean percentage of “left” responses for Vernier Trials during Experiment 2. This figure illustrates the mean percentage of “left” responses given by the participants at each of the possible three Vernier Target display locations (left, center, and right). Error bars represent the standard error of the mean.

As before, the percentage of “left” responses was computed for each participant for each combination of Cue Location (left or right) and Vernier Target (left, center, or right). These data were analyzed with a 2 (Cue Location) × 3 (Vernier Target) repeated-measures analysis of variance (ANOVA). There was an effect of Vernier Target [*F*(2, 50) = 310.24, *MSE* = 271, *p* < 0.001, ${\text{η}}_{\text{G}}^{2}$ = 0.8512]. Participants were able to distinguish the Vernier Targets. Contrary to the results obtained from Experiment 1, there was an effect of Cue Location [*F*(1, 25) = 8.746, *MSE* = 247, *p* = 0.0067, ${\text{η}}_{\text{G}}^{2}$ = 0.0682], with responses generally tending in the direction opposite the cue. This effect was qualified by the interaction [*F*(2, 50) = 3.915, *MSE* = 72, *p* = 0.0263, ${\text{η}}_{\text{G}}^{2}$ = 0.01875], with this bias being most pronounced when there was no offset between the two lines of the Vernier stimulus (see Figure 6). For central Vernier stimuli, left responses were more common following rightward (52.45%) than leftward cues (39.64%); [*t*(25) = 2.776, *p* = 0.0103, *M* = 12.81%, 95%CI = -22.31 – 3.31%]. Furthermore, a cross-experiment analysis on the Vernier Targets revealed a significant interaction between Cue Location and Experiment [*F*(1, 50) = 6.296, *MSE* = 143, *p* = 0.0154, ${\text{η}}_{\text{G}}^{2}$ = 0.0156], indicating that our cues led to an ARE in Experiment 2 but not in Experiment 1.

The results from Experiment 2, for the first time, demonstrate an ARE in the context of temporal evidence for involuntary orienting. The results also suggest that the absence of an ARE in Experiment 1 had little to do with the intermixing of Vernier and attention targets. Most importantly, contrasting the present results with the results of Experiment 1 provides reasonably strong evidence that the ARE depends critically on peripheral cues, less so on attentional orienting more generally.

## General Discussion

In finding the original repulsion effect, Suzuki and Cavanagh (1997) concluded the effect depends on attentional mechanisms, and named it the Attentional Repulsion Effect. Following this study, every subsequent study has implicitly assumed an attentional underpinning and has used transient, peripheral sensory stimuli, mainly in the form of brief onset-offset cues (e.g., Pratt and Turk-Browne, 2003; Pratt and Arnott, 2008; Kosovicheva et al., 2010; Fortenbaugh et al., 2011). The current study was designed specifically to address whether attention is sufficient in and of itself, without peripheral cues, for generating an ARE. To do this, in Experiment 1, we guided attention to the visual periphery with spatially informative number cues at fixation (e.g., Rizzolatti et al., 1987; Ristic and Kingstone, 2006). In Experiment 2, we used the standard spatially uninformative peripheral onset-offset cues. In both experiments, we verified that the cues oriented attention by taking chronometric measurements while also assessing for the presence of an ARE. We found that while both cue types led to similar RT advantages at cued locations, only the peripheral cues led to AREs. The presence of an ARE in combination with the standard temporal evidence of orienting in Experiment 2 rules out the possibility that intermixing Vernier and Attention targets created a dual-tasking scenario disfavoring the ARE in Experiment 1. Collectively, the findings demonstrate that attention in and of itself may not be sufficient for generating the ARE and suggest that some sort of sensory event in the periphery is necessary.

The results of the current study appear to conflict with the conclusion reached by Suzuki and Cavanagh (1997), namely that the ARE is exclusively dependent on attention. However, as Suzuki and Cavanagh made use of transient peripheral cues in both their involuntary and voluntary attentional experiments, they were unable to determine the extent to which the ARE relied on these peripheral cues. Specifically, any experimental procedure that incorporates sensory stimulation in the periphery prior to the presentation of the Vernier stimulus cannot disentangle the role of attention from the role of peripheral sensory stimulation in generating a repulsion effect. That is, any repulsion effect attributed to attentional mechanisms may instead be attributed, at least in part, to sensory interactions. The current study circumvented this issue altogether by incorporating a central predictive cue to guide attention endogenously, in the absence of any sensory stimulation in the periphery.

In interpreting the results obtained from the current study, it is worth considering the possibility that central cueing, but not peripheral cueing, leads to a uniform distribution of attention over the entire left or right hemifield and thus results in a lack of an ARE. The literature on attentional gradients however indicates that this possibility is very unlikely. In a seminal study, Rizzolatti et al. (1987) investigated the distribution of attention from central, predictive number cues within and between hemifields. The authors found that RTs were fastest when the target occurred precisely at the cued location and that RTs tended to increase as a function of the distance between the cued location and target location. RTs increased considerably whenever the target was presented in a different hemifield than that which was cued (i.e., across the vertical or horizontal meridian). In addition, Dori and Henik (2006) further demonstrated that fairly fine-tuned endogenous attentional gradients exist within each visual quadrant. Moreover, there are no studies that refute these findings. Thus, there is every reason to believe that endogenous attention is as spatially distinct as exogenous attention.

In addition Arnott and Goodale (2006) were able to elicit an ARE from peripheral auditory cueing (i.e., using lateralized sounds). While evidence suggests that both endogenous and exogenous auditory cueing lead to location-specific attentional effects, these effects are generally stronger with visual than auditory cues (Spence and Driver, 1994; Rorden and Driver, 2001). Taken in the context of the findings of Rizzolatti et al. (1987) and Dori and Henik (2006), the implication is that central visual cues would be even more likely to orient attention to a specific location than peripheral auditory cues. As exogenous auditory cues are yet capable of generating an ARE, it seems unlikely that the lack of an ARE observed in Experiment 1 can be explained by a lack of attentional focus at the cued location. Furthermore, in our study, symbolic orienting was clearly precise enough to generate a comparable RT advantage for valid over invalid trials in both Experiments 1 and 2, with only the latter showing an ARE.

Another aspect worth considering is the difference in CTOAs between experiments. Specifically, the interval between the endogenous cue and the Vernier target was longer (500 ms) than the interval between the exogenous cue and target (150 ms). This difference was deliberate, as peripheral cues have been shown to orient attention more rapidly than central cues (Jonides, 1981). Accordingly, it was necessary for the CTOA to be long enough for endogenous orienting to build up in order for an ARE to be detected, should one exist (Suzuki and Cavanagh, 1997). Furthermore, Suzuki and Cavanagh obtained complete CTOA curves for both their involuntary attention and voluntary attention conditions. Importantly, they found that AREs were still apparent at CTOAs well past 800 ms. Thus, it seems unlikely that the CTOA difference across our two experiments could account for the observed results. Nevertheless, additional studies exploring the effects of varying CTOAs when employing central predictive cueing paradigms would be beneficial in order to fully establish the limits to this parameter of the ARE. In addition, considering the fact that DiGiacomo and Pratt (2012) did not observe an ARE when they presented the cue and target to different eyes, it would also be of value to investigate eye movements in the context of the ARE when presenting central and peripheral cues.

Yet, while the dual-task paradigm implemented in our experiments for the first time allows for the empirical measurement of attentional orienting within the context of assessing for an ARE, it also introduces a limitation to our study in the form of unequal attentional processing load across the two experiments. That is, whereas the peripheral cue in Experiment 2 did not require any form of interpretation, the central cue in Experiment 1 (which consisted of the symbolic numbers “2” and “5”) did. It is possible that the additional attentional load introduced by the necessity to interpret the central cue in Experiment 1 could underlie, at least in part, the lack of an observed ARE in this experiment. This possibility rests on the assumption that the ARE behaves differently from RT in response to increased attentional demands, as no observable difference in RT could be detected across the two experiments. The findings by Pratt and Arnott (2008) demonstrate that the ARE behaves as a spatial analog to RT in response to (a) single onset, offset, and onset-offset cues; (b) simultaneous onset and offset displays; and (c) pop-out color cue displays. It is not clear why the difference in attentional load across our experiments should succeed in differentiating the behavior of the ARE from that of RT when experimental manipulations contrasting the response of the two to various cue types and displays have failed to accomplish this task in the past. Even though load is an issue worth exploring, it remains clear from the present work that it is possible to obtain attentional orienting effects and have no corresponding ARE.

Finally, although our findings suggest that attention without peripheral sensory stimulation may not be sufficient for generating an ARE, it remains possible that attention modifies the magnitude of the ARE when generated by stimulation in the visual periphery. This notion is supported by the report by Pratt and Arnott (2008), namely that the ARE behaves as a spatial analog to RT in that it mirrors the results obtained from chronometric attentional tasks. Furthermore, Gozli and Pratt (2012) reported a smaller ARE with transient peripheral cues that mismatched an implemented attentional control set, as compared to peripheral cues that matched said control set. Thus, while not critically dependent on attentional mechanisms as much as on peripheral cues, it seems that the ARE can be altered by attention. As such, it might be more accurate to describe the ARE as a spatial misperception generated by low-level sensory interactions, which can be modified by attention. Further research examining this proposal is needed to help clarify the limits of the ARE.

## Data Availability Statement

The raw data supporting the conclusions of this manuscript will be made available by the authors, without undue reservation, to any qualified researcher.

## Ethics Statement

This research was approved by the University of Toronto Research Ethics Board.

## Author Contributions

MH, JP, and AP contributed to the design of the study and to the subsequent drafts. MH wrote the Python script for the experiment and analyzed the data. AP wrote the first draft of the manuscript. All authors have read and approved of the submitted version.

## Conflict of Interest Statement

The authors declare that the research was conducted in the absence of any commercial or financial relationships that could be construed as a potential conflict of interest.

## Footnotes

1 The mean cueing effect for the excluded participant was 340 ms (valid *RT* = 875 ms and invalid *RT* = 1216 ms), which was 4.2 standard deviations greater than the group mean. We figured that this participant was idiosyncratic and that including them would lead to an overestimation of the mean cueing effect and variance. If this person is included in the analysis, the M cueing effect is 31.16 ms [*t*(26) = 2.24, *p* = 0.034, 95%CI = 2.58 – 59.74 ms]. With or without this person, the cue did not generally produce an ARE (*F* < 1).

## Abbreviations

ARE: Attentional Repulsion Effect
CTOA: cue-target onset asynchrony
ms: milliseconds
RT: reaction time
SNARC: spatial-numerical association of response codes
V1: primary visual cortex
V2: secondary visual cortex/visual area V2.
